# Cervical Whiplash Syndrome: A Case Report of a Work Accident

**DOI:** 10.7759/cureus.33742

**Published:** 2023-01-13

**Authors:** Maria José Costa de Almeida, Luís Almeida, Moreira Freire Duarte, Sara Alvez de Matos

**Affiliations:** 1 Medicine, Centro Hospitalar Universitário do Porto (CHUP), Porto, PRT; 2 Pharmacology & Therapeutics, Faculdade de Medicina da Universidade de Coimbra (FMUC), Coimbra, PRT; 3 Serviço de Saúde Ocupacional (SSO), Centro Hospitalar Universitário do Porto (CHUP), Porto, PRT; 4 Serviços de Saúde, Higiene e Segurança do Trabalho (SSHST), Centro Hospitalar do Tâmega e Sousa (CHTS), Porto, PRT

**Keywords:** post-traumatic bodily injury, whiplash syndrome, cervical whiplash syndrome, work disability, muscle atrophy, cervicobrachialgia, occupational accident, whiplash

## Abstract

Cervical whiplash syndrome (CWS) or whiplash syndrome is a highly debated concept because there is still no consensus on its definition -- symptoms are usually very severe but the pain’s root cause is typically uncertain. Clinical investigation and detailed radiology seldom identify a specific pathology. Thus, soft tissue injury is generally considered the most likely explanation for the symptoms, although it is difficult to confirm, even by MRI. We describe the clinical case of a physical education teacher who suffers an accident in one of her classes. The following day she is assessed at the emergency department and, after undergoing a radiological study of the cervical spine, she is diagnosed with straight cervical spine (kyphosis). She is observed again seven days later due to persistent pain but sent home with unchanged indications for rest and medication. After that her cervicobrachialgia progressively worsens, limiting her left shoulder active mobility and leading to associated muscle atrophy (in addition to a burnout syndrome). Several years after she is considered to have a total permanent disability. Finally, the authors propose that CWS should be approached according to the post-traumatic bodily injury evaluation methodology, suggesting some interventions.

## Introduction

The cervical whiplash syndrome (CWS) or whiplash syndrome is a traumatic injury that occurs due to a cervical hyperextension-hyperflexion which affects the soft tissue structures that surround the cervical spine [1]. Clinical investigation or detailed radiology rarely identify a specific pathology. Brachial plexus involvement has been confirmed in some patients with chronic whiplash [2] but the actual incidence of symptoms associated with chronic disability is not known [3-4]. Symptoms can take hours or even days to appear. The most common are neck pain (88%-100%) and headache (54%-66%), but low back pain and/or other back pain, fatigue, dizziness, paresthesias, nausea, and jaw pain may occur. Many patients also report anxiety, depression, and lack of concentration [5-6]. The prognosis of CWS is generally favorable [3], but there is great variability in terms of frequency, severity, and duration of the disability. This variability partially depends on each country's tradition in litigation and compensation [7], but this cannot explain all the differences, specially within the same population. Two systematic reviews [3-4] found little consistency in the factors influencing the outcome, such as age, pre-existing psychological problems, and anxiety. However, they did consider pain severity, headache, and functional limitations as major prognostic factors.

## Case presentation

**Figure 1:**
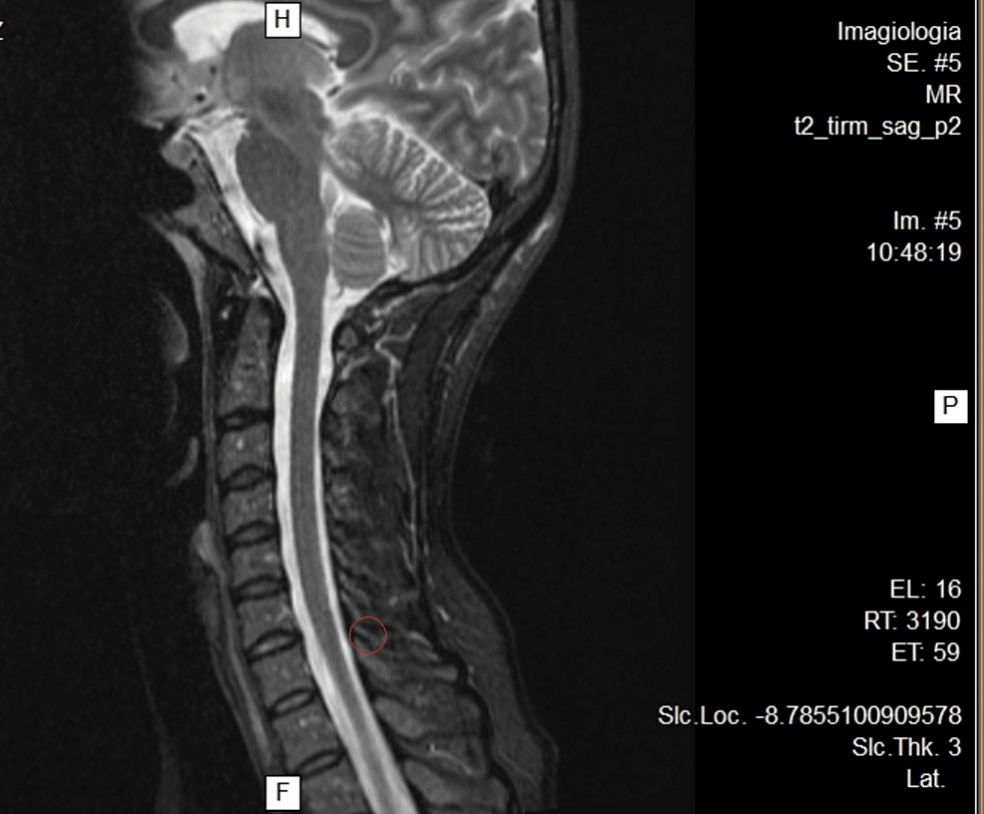
Cervical MRI. Incipient foraminal asymmetry in C4-C5

**Figure 2:**
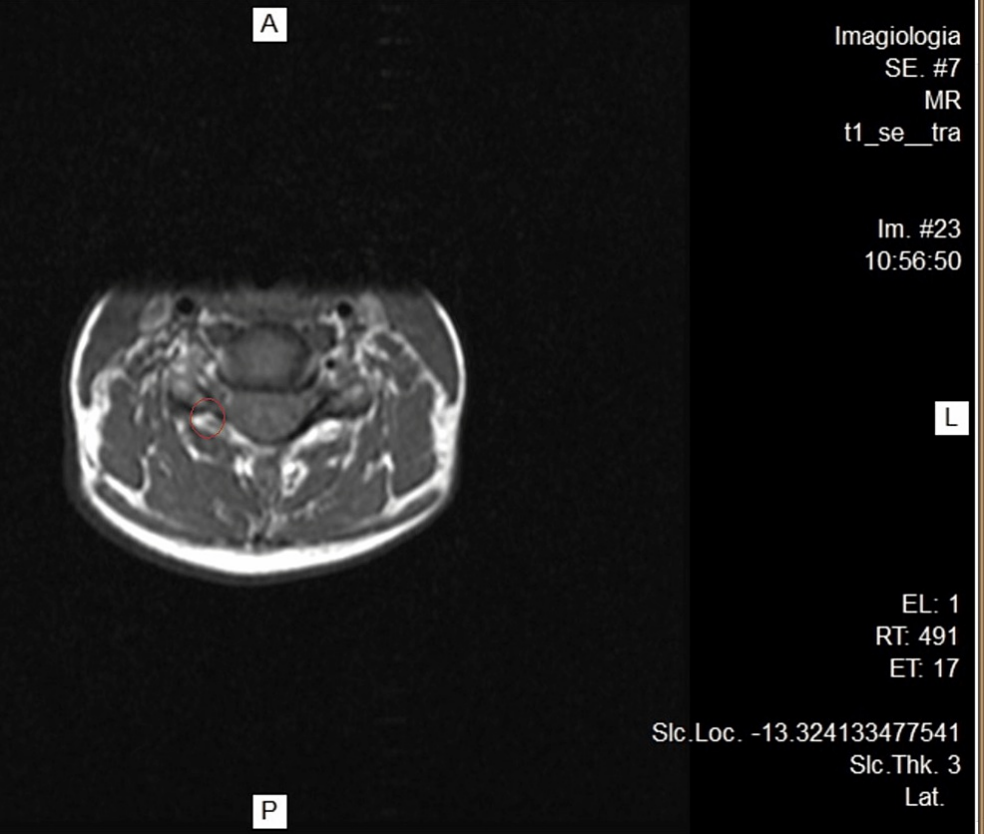
Cervical MRI. C5-C6, with less expression on the left

**Figure 3:**
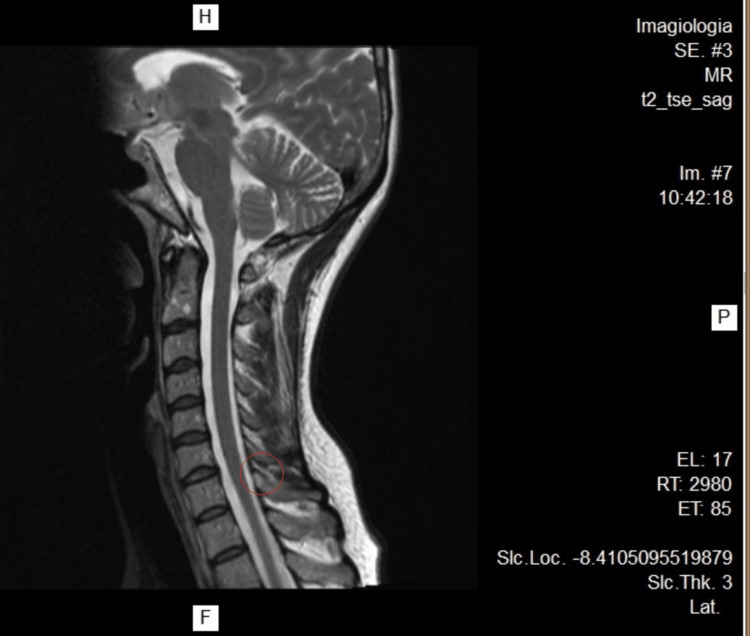
Cervical MRI. Without stenotic features

A 45-year-old female patient, physical education teacher, suffered a blow to the cervical region while helping her student do a handstand and got hit by the student's legs on that area and left shoulder. Immediately, she felt a major snap in her neck, a shooting pain that ran through the entire thoracic spine and momentary loss of vision and hearing. She underestimated the event; as her muscles were still warm and she took it as a normal incident in her profession. As symptoms worsened, the following day she went to the emergency department (ED) and got a diagnosis of cervical rectification based on the cervical X-ray. She was discharged with medication (diclofenac 75 mg/3 mL IM + paracetamol 500 mg/thiocolchicoside 4 mg/2 mL IM for 6 days) and without work limitations. As the condition continues to worsen (she can hardly get out of bed due to the pain), she goes to the ED after 7 days and was diagnosed with a cervical sprain, rhomboid rupture, and various muscle contractures. She went for 10 months without working, as she underwent several sessions of physiotherapy, mesotherapy, acupuncture, kinesiotherapy, thermal treatment, psychotherapy, pain consultation, psychiatry (with a diagnosis of anxiety-depressive disorder with somatization in the context of post-traumatic stress), and different combinations of pharmacological drugs to minimize the pain, physical weakness, and sleep disturbance. Despite all the efforts, the accumulation of fatigue culminates in a burnout episode (emotional, physical, and psychological exhaustion). Throughout this period, the patient was presented to numerous medical committees to assess her work inability and legally define the event as a work accident. Without clinical improvement, she performed a cervical MRI that described at intersomatic levels, in C4-C5, incipient foraminal asymmetry (Figure 1), which is repeated in C5-C6 with less expression on the left (Figure 2); without stenotic features (Figure 3). She made an electromyography (EMG) that refers moderate neurogenic alterations in the muscles dependent on the bilateral C5 and C6 myotome on the left side, to be assessed as some degree of cervical radicular suffering. She repeats a cervical X-ray that maintains the diagnosis of cervical rectification and performs a cervical CT that describes signs of rectification of the physiological cervical lordosis in the examination position. Ultrasonography of the left shoulder excludes rotator cuff tendinopathy; it was performed due to left cervicobrachial pain irradiating to the third and fourth fingers of the hand with associated paresthesia, suspicion of left shoulder tendinopathy, and parathoracic pain on the same side.

Currently, she presents functional limitations (Table 1), disabling sequelae with recurrence of mixed left cervicobrachialgia (musculoskeletal and neuropathic), in the context of a structured antalgic vicious posture that conditioned the development of cervical, scapular, paradorsal and left upper limb muscle atrophy and proprioceptive deficit. Current treatment: Sertraline 50 mg 1 id + amitriptyline 25 mg, 1/2 id + trazodone 150 mg 1/3id + Diazepam 5 mg, 1 id + Flupirtine 100 mg id. Known drug allergy: ranitidine and tramadol. The total permanent disability to perform her job was declared in the court of labor.

**Table 1:** Cervical mobility examination and upper limb tests. *with pain radiating along the posterolateral edge of the left arm to the elbow; **with pain radiating to the hand

Cervical and brachial plexus mobility		
Cervical spine	Flexion	20º
	Extension	14º
	Right-lateral tilt	14º
	Left-lateral tilt	6º
	Right rotation	36º
	Left rotation	30º *
Left arm (vs. Right)	Abduction	65º (vs. 180º)
	Supination	35º ** (vs. >180º)
	Antepulsion	90º (vs. 180º)
	Retropulsion	33º (vs. 60º)
	Pain and daily paresthesias in the dermatomes C3, C4, D1 a D7	

## Discussion

The majority of whiplash injuries progress positively, only a few cases became chronic with poor response to treatment. The variety and intensity of complaints is initially unexpected but becomes clear after a few hours. With the appropriate rest, physical and pharmacological support one can expect a resolution within days or a few months. However, there are a small number of cases in which the condition is chronic, with a poor response to treatment, little or no visibility in imaging tests, but exuberant functional disability. The present report case is an example of this -- vertebral trauma in flexion and forced lateralization, with involvement of ligamentous structures and stretching of the left cervical roots. The pain’s intensity, fueled by the physical effort of the daily activities, combined with the functional limitations conditioned the decision to declare the total inability to teach physical education.

On top of the above, an assessment of body damage in the context of CWS can serve as a key data set to identify a defining core of the syndrome and its subdivisions, as cross-sectional information and miscellaneous data are collected for all patients. The authors propose that CWS should be addressed according to the post-traumatic bodily injury assessment methodology, suggesting the following interventions: (a) periodic assessment of pain, while studying its characteristics, intensity, irradiation, analgesic and aggravating maneuvers, periodicity, relationship with daily activities and repeated movements, effects of pharmacological and non-pharmacological therapies, etc.; (b) periodic evaluation of mobility, while studying the cervical spine, dorsal-lumbar, upper limbs, and relating them to the characteristics mentioned in the previous paragraph; (c) regular assessment of muscle strength, studying it at the cervical level, dorsal-lumbar spine and upper limbs, also relating them to other characteristics; (d) evaluation of pericervical structures, through imaging, in functional terms, by combining painful points, mobility deficits, functional tests, response to treatments, physical or pharmacological, response to stimulation, combining all these and other data with the other suggested characteristics.

## Conclusions

After an accident, whether at work or of any other nature (for example, traffic), the assessment of CWS, as repairable bodily damage, is a very useful tool in the study of this clinical entity because it makes it possible to separate the majority of clinical cases in which the discomfort ceases in short period of time, from the minority in which the lesions become chronic. Also, it gives medical relevance to a minority of clinical cases currently overlooked because they have non-specific and/or tenuous imaging most of the time. The follow-up of the clinical case and the study of CWS need multidisciplinary on the diagnostic and/or therapeutic interventions because CWS combines subjective damage, such as pain and paresthesia, with objective damage, such as functional deficits and muscle strength. It combines direct damage, more attributable to cervical injuries, with indirect, more comprehensive damage, with more distant cervical causes, such as burnout, incapacity for work or daily life activities, personality disorders, or psychiatric disorders.
